# Bioinformatics Approach to Investigating the Immuno-Inflammatory Mechanisms of Periodontitis in the Progression of Atherosclerosis

**DOI:** 10.3390/cimb47030197

**Published:** 2025-03-17

**Authors:** Wenling Yang, Jianhua Xie, Xing Zhao, Xuelian Li, Qingyi Liu, Jinpeng Sun, Ruiyu Zhang, Yumiao Wei, Boyuan Wang

**Affiliations:** 1 Department of Cardiology, Union Hospital, Tongji Medical College, Huazhong University of Science and Technology, Wuhan 430022, China; d202382084@hust.edu.cn (W.Y.); tjmedxjh@163.com (J.X.); lxl02914121@163.com (X.L.); liuqingyizzu@163.com (Q.L.); sunsunjinjin@163.com (J.S.); zrydoctor@163.com (R.Z.); 2 Hubei Key Laboratory of Biological Targeted Therapy, Union Hospital, Tongji Medical College, Huazhong University of Science and Technology, Wuhan 430022, China; 3 Hubei Provincial Engineering Research Center for Immunological Diagnosis and Therapy of Cardiovascular Diseases, Union Hospital, Tongji Medical College, Huazhong University of Science and Technology, Wuhan 430022, China; 4 Department of Critical Care Medicine, Union Hospital, Tongji Medical College, Huazhong University of Science and Technology, Wuhan 430022, China; zx030310@163.com

**Keywords:** atherosclerotic plaque, periodontitis, stroke, bioinformatics analysis

## Abstract

Unstable atherosclerotic plaques are a major cause of acute cardiovascular events and ischemic stroke. Clinical studies have suggested a link between periodontitis and atherosclerotic plaque progression, but the underlying mechanisms remain unclear. To investigate this, transcriptomic datasets related to periodontitis and atherosclerosis were downloaded from Gene Expression Omnibus. A weighted gene co-expression network analysis was used to identify gene modules associated with periodontitis, and the Limma R package identified differentially expressed genes (DEGs) between unstable and stable plaques. Overlapping genes were defined as periodontitis-related DEGs, followed by functional enrichment analysis and protein–protein interaction network construction. Machine learning methods were used to identify biomarkers for unstable plaques related to periodontitis, which were validated using external datasets. Immune infiltration and single-cell analyses were performed to explore the relationship between biomarkers and immune cells. A total of 161 periodontitis-related DEGs were identified, with the pathway analysis showing associations with immune regulation and collagen matrix degradation. *HCK*, *NCKAP1L*, and *WAS* were identified as biomarkers for unstable plaques, demonstrating a high diagnostic value (AUC: 0.9884, 95% CI: 0.9641–1). Immune infiltration analysis revealed an increase in macrophages within unstable plaques. Single-cell analysis showed *HCK* expression in macrophages and dendritic cells, while *NCKAP1L* and *WAS* were expressed in macrophages, dendritic cells, NK cells, and T cells. Consensus clustering identified three expression patterns within unstable plaques. Our findings were validated in atherosclerotic mouse models with periodontitis. This study provides insights into how periodontitis contributes to plaque instability, supporting diagnosis and intervention in patients with periodontitis.

## 1. Introduction

Atherosclerosis is a major cause of cardiovascular and cerebrovascular diseases, leading to high morbidity and mortality worldwide [1]. Research has confirmed that the formation and progression of atherosclerotic plaques involve complex immune and inflammatory responses within a highly intricate physical and biochemical microenvironment, comprising various cell types and molecules, including immune cells, endothelial cells, inflammatory factors, and chemokines [2,3]. Atherosclerotic plaques are typically classified into stable and unstable types based on their phenotype [4]. Stable plaques are often characterized by calcification and a complete fibrous cap or endothelial layer, while unstable plaques are formed by lipid accumulation, neovascularization, thrombosis, and connective tissue protruding into the vessel lumen, making them softer, prone to rupture, and susceptible to internal bleeding [5,6]. The instability and rupture of unstable plaques can lead to severe myocardial infarction and ischemic stroke [7]. Therefore, identifying and investigating biomarkers and potential therapeutic targets for unstable atherosclerotic plaques is crucial for preventing adverse cardiovascular and cerebrovascular events [8].

Periodontitis, a chronic inflammatory disease affecting the soft and hard tissues surrounding teeth, is the sixth most common disease globally, with a prevalence of 45–50% among the population [9]. Periodontitis is a chronic inflammatory disease initiated by dental plaque biofilm, with its core mechanisms involving the interplay between immune–inflammatory responses and pathogenic bacteria [10]. Pathogenic bacteria within the dental plaque, such as *Porphyromonas gingivalis* and *Aggregatibacter actinomycetemcomitans*, release virulence factors, including lipopolysaccharides and proteases [11]. These factors activate the host immune system, prompting gingival epithelial cells and immune cells (e.g., macrophages, neutrophils) to produce a plethora of pro-inflammatory cytokines, such as IL-1β, TNF-α, and IL-6 [11]. These inflammatory mediators further exacerbate the inflammatory response in the gingival and periodontal tissues, leading to tissue destruction and bone resorption. Concurrently, pathogenic bacteria evade immune clearance and disrupt the host defense mechanisms, intensifying the inflammatory response and creating a vicious cycle that ultimately results in irreversible damage to the periodontal supporting tissues [12].

Recent studies have confirmed periodontitis as an independent cardiovascular risk factor, correlating not only with the prevalence of atherosclerosis but also with the progression of atherosclerotic plaques [13]. One study demonstrated a positive correlation between periodontitis and carotid intima–media thickness [14]. Another study indicated that patients with periodontitis have a higher risk of acute cardiovascular events, such as myocardial infarction [15]. These findings suggest that periodontitis plays a significant role in the progression and destabilization of atherosclerotic plaques.

Periodontitis can introduce bacteria into the bloodstream, leading to localized inflammation in atherosclerotic plaques and increasing systemic chronic inflammation [16,17]. This process activates various inflammatory cells in the bloodstream through mechanisms of trained immunity, which can then infiltrate affected areas and promote distal inflammatory disease, such as atherosclerosis [18]. However, the exact mechanisms, related pathways, and biomarkers through which periodontitis leads to the destabilization of atherosclerotic plaques remain unclear [19]. In this study, we hypothesize that chronic periodontitis may promote the progression of atherosclerotic plaques through immune–inflammatory mechanisms, leading to plaque destabilization and subsequent cardiovascular events. With advancements in chip sequencing and high-throughput sequencing technologies, our understanding of disease-related gene expression patterns and local cellular infiltration has significantly improved [20]. The application of machine learning methods aids in the identification and characterization of molecular and cellular biomarkers related to disease diagnosis and treatment [21]. In this study, we utilized transcriptomic data from the Gene Expression Omnibus (GEO) public database to explore the potential molecular mechanisms by which periodontitis-related genes contribute to atherosclerotic plaque instability. We also identified the expression patterns of biomarkers associated with unstable plaques related to periodontitis, their relationship with immune cells, and potential therapeutic and regulatory targets. This research may provide new insights for the diagnosis and treatment of unstable atherosclerotic plaques in patients with periodontitis.

## 2. Materials and Methods

### 2.1. Data Source

**Figure 1:**
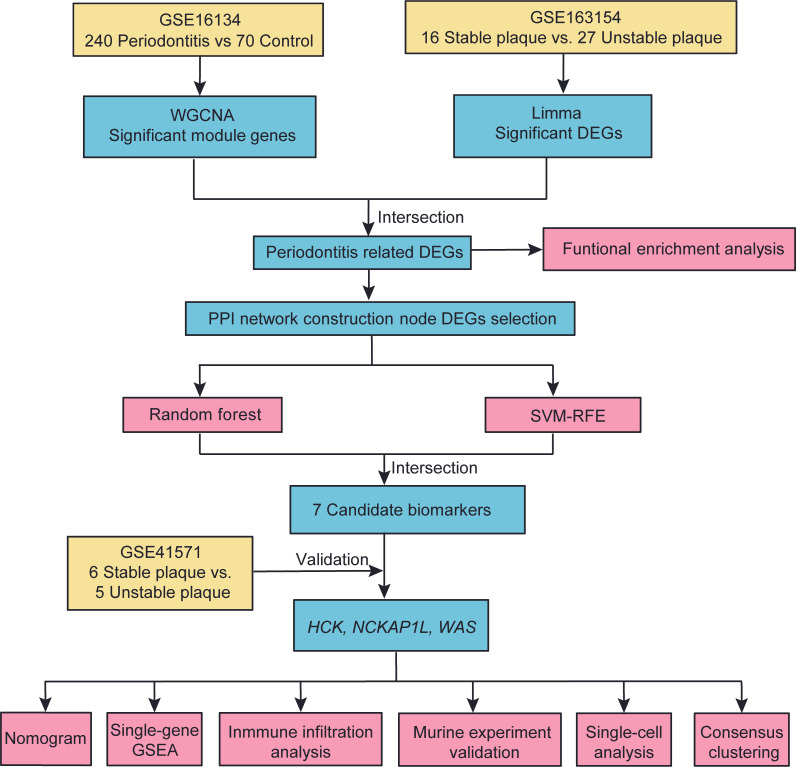
Workflow of this study. DEGs, differentially expressed genes; WGCNA, weighted genes co-expression network analysis; PPI, protein–protein interaction; SVM-RFE, support vector machine recursive feature elimination; GSEA, gene set enrichment analysis.

The flowchart of this study is shown in Figure 1. All public data used in the research were sourced from the GEO database. The GSE16134 dataset, released in 2009, comprises samples from 120 patients with periodontitis, including gingival lesions and healthy areas, which were analyzed using microarray detection on the GPL570 platform [22]. A total of 120 patients undergoing periodontal surgery were included in this study. From each patient, at least two interproximal gingival papillae (ranging from 2 to 4) were collected from the maxillary posterior region. Prior to tissue collection, subgingival plaque samples were obtained from both the mesial and distal aspects of each papilla. The gingival tissue samples were then processed for RNA extraction, followed by reverse transcription and labeling. The labeled cDNA was subsequently hybridized to whole-genome microarrays (a total of 310 arrays). The GSE163154 dataset, published in 2020, includes microarray sequencing of 16 stable and 27 unstable carotid plaques, detected using the GPL6104 platform [23]. The database includes both stable and unstable plaques, which were carefully distinguished and sampled as follows: Using laser microdissection, we isolated total RNA from macrophage-rich regions of stable and ruptured human atherosclerotic plaques obtained from carotid endarterectomy specimens. The classification of plaques as stable or unstable was based on a comprehensive characterization using clinical, radiological, and histological criteria. Following this classification, RNA from the macrophage-rich regions of each plaque type was separately extracted, processed, and subjected to whole-genome gene expression profiling using microarrays. Additionally, the validation dataset GSE41571 consists of microarray sequencing data from 6 stable and 5 unstable carotid plaques, also using the GPL570 platform [24]. We also selected single-cell mRNA sequencing data from 6 unstable plaques in the GSE253904 dataset to analyze the expression of biomarkers in specific cell populations [25]. Detailed information regarding each dataset is provided in Table 1.

### 2.2. Normalization and Annotation of Microarray Data

Public transcriptome datasets were downloaded using the GEOquery R package [26]. The probe expression matrices were then converted to gene expression matrices using the platform annotation files from GEO. Normalization of the probe expression matrices was performed using the Robust Multi-Array Average method. For genes represented by multiple probes, the average expression level of those probes was calculated.

### 2.3. Weighted Gene Co-Expression Network Analysis (WGCNA)

WGCNA is an advanced bioinformatics method used to analyze gene expression patterns across multiple samples, aiming to identify gene expression modules associated with clinical traits [27]. In this study, we clustered genes with similar expression patterns into modules and analyzed their associations with periodontitis. Initially, we selected the top 5000 genes with the highest median absolute deviation from the periodontal disease-related expression matrix. The expression matrix was filtered using the goodSamplesGenes function to exclude low-quality genes and samples. The soft-thresholding power (β) was determined from the co-expression similarity matrix to establish adjacency relationships, which were then transformed into a Topological Overlap Matrix (TOM) to derive gene ratios and their associated dissimilarities. Hierarchical clustering was performed to group genes into distinct modules, with each module representing a set of co-expressed genes. The dynamicTreeCut function was employed for visualization. Using TOM-based dissimilarity measures, we conducted average linkage hierarchical clustering with a minimum gene count of 50, clustering highly correlated genes into modules, represented as a gene dendrogram. Module feature genes were evaluated for similarity (similarity > 0.75) to merge comparable modules. Finally, we generated a heatmap showing the correlation between gene expression modules and periodontal disease. Gene modules with a correlation coefficient > 0.3 and a *p*-value < 0.05 were defined as periodontal disease-related modules and were used for subsequent analyses.

### 2.4. Selection of Differentially Expressed Genes (DEGs)

DEGs between stable and unstable atherosclerotic plaques were identified using the Limma R package [28]. The thresholds for defining DEGs were set as an adjusted *p*-value of less than 0.05 and an absolute log2 fold change (FC) greater than 1.

### 2.5. Functional Enrichment Analysis

Functional enrichment analysis of the periodontitis-related DEGs was performed using the Gene Ontology (GO), Kyoto Encyclopedia of Genes and Genomes (KEGG), and Reactome databases. The results were analyzed and visualized using the clusterProfiler R package [29], with a threshold of a *p*-value < 0.05 to identify the top ten most significant pathways

### 2.6. Protein–Protein Interaction (PPI) Network Establishment

The STRING database has become an indispensable tool in PPI analysis [30]. It vividly illustrates the interactions between proteins through network diagrams, providing insights into the intricate regulatory networks within cells. The PPI network of periodontitis-related DEGs was analyzed using the STRING database, with a minimum interaction score threshold of 0.7. The CytoHubba plugin of Cytoscape software (3.8.0) was utilized to identify the top 30 hub genes ranked by the Degree algorithm.

### 2.7. Machine Learning for Biomarker Selection and ROC Curve Construction

To further identify biomarkers for unstable plaques related to periodontitis from the node genes, we employed various machine learning algorithms, including support vector machine–recursive feature elimination (SVM-RFE) and random forest. The intersection of the gene sets from both methods was taken as candidate biomarkers. ROC curves were constructed using R package pROC, and the area under the curve (AUC) was calculated to evaluate the diagnostic efficacy of the selected potential biomarkers [31].

### 2.8. Nomogram Construction and Single-Gene Gene Set Enrichment Analysis (GSEA)

Candidate biomarkers with an AUC > 0.7 and showing significant expression in both the discovery and validation datasets were selected as biomarkers for unstable plaques related to periodontitis. The nomogram for diagnosis was constructed using the rms package in R. A single-gene GSEA of the biomarkers was performed based on the GO database using the clusterProfiler package in R, with the most significant five enriched pathways for each biomarker displayed under the criterion of a *p* value < 0.05 [29].

### 2.9. Immune Infiltration Analysis

Immune infiltration analysis was used to assess the degree of immune cell infiltration in pathological tissues, aimed at understanding the presence and distribution of immune cells within the tissue. In this study, we utilized the CIBERSORT package in R, which employs linear support vector regression to deconvolute the expression matrix of atherosclerotic plaques, enabling us to evaluate the infiltration of immune cells [32]. We also compared the expression levels of biomarkers with the relative abundance of immune cells.

To further analyze the abundance of immune and stromal cells within the plaques, we employed the ESTIMATE package in R and conducted correlation analyses with the expression of the identified biomarkers [33].

### 2.10. Single-Cell RNA Sequencing Data Analysis

The dataset GSE253904 contains single-cell mRNA sequencing data from six unstable carotid atherosclerotic plaques. We processed the data using the Seurat package in R and applied the Harmony package to mitigate batch effects. The expression information of 20,687 cells were obtained, which were visualized using t-SNE for dimensionality reduction to illustrate the identified cell subpopulations. Then we utilized the FindAllMarkers function to identify and filter characteristic genes for each cell type, applying thresholds of a >25% expression across cells and a log2FC > 0.25. Finally, we annotated the characteristic genes and cell subpopulations using CellMarker 2.0 [34].

### 2.11. Consensus Clustering

Consensus clustering is an unsupervised clustering approach aimed at identifying potential expression subtypes within unstable plaques. We utilized the ConsensusClusterPlus package in R along with the K-means clustering algorithm, selecting the top 5000 genes with the highest median absolute deviation for clustering and computing a similarity matrix. The similarity matrices from multiple clustering iterations were integrated using an averaging method to derive a consensus matrix, resulting in the final clustering outcomes. The gene expression profiles for each disease subgroup were visualized using principal component analysis (PCA) plots. We then compared the expression levels of the identified biomarkers across the different subtypes.

### 2.12. Establishment of Atherosclerosis Mouse Model

Male APOE^−/−^ C57BL/6 mice, aged 5 weeks, were purchased from Beijing Vital River Laboratory Animal Technology Co., Ltd. (Beijing, China), and housed in ventilated cages under specific-pathogen-free (SPF) conditions at the Animal Experiment Center of the Tongji Medical College, Huazhong University of Science and Technology, Wuhan, China. After one week of acclimatization on a regular diet, the mice were fed a high-fat diet (21% fat, 0.15% cholesterol) for 12 weeks to induce atherosclerotic lesions, while being maintained under a strict 12 h light/dark cycle. Ethical approval for animal-related procedures was granted by the Animal Care and Use Committee of Huazhong University of Science and Technology ([2023] IACUC Number: 4168), and all protocols were performed in accordance with the guidelines set by the National Institutes of Health.

### 2.13. Establishment of Chronic Periodontitis Mouse Model

Ligature-induced periodontitis simulates human periodontitis by creating a local environment conducive to biofilm retention, thereby inducing inflammation and bone loss [35]. To investigate the impact of experimental periodontitis on the progression of atherosclerotic plaques, a chronic periodontitis model was established in mice as previously described after 12 weeks of high-fat diet feeding [18]. Briefly, mice were initially anesthetized with 4% (*v*/*v*) isoflurane. Once they showed difficulty standing, anesthesia was maintained by delivering 2% (*v*/*v*) isoflurane via a face mask. The adequacy of the anesthesia was confirmed by the absence of a response to gentle toe pinching. Then, 5-0 silk sutures were ligated around the left and right maxillary second molars in the experimental group, while the corresponding teeth in the control group were left unligated. To alleviate pain, buprenorphine was administered subcutaneously every 12 h for 48 h post gingival ligation surgery. After 21 days, the mice were euthanized. Euthanasia was performed with an intraperitoneal injection of excessive pentobarbital. After a few minutes, once the mice were confirmed to have succumbed due to central nervous system suppression, specimen processing was performed. Peripheral blood was collected, and the aortas were carefully excised under a microscope for subsequent experiments and analyses.

### 2.14. Immunohistochemistry (IHC) Experiment

The process of the immunohistochemical staining of HCK in the mouse aorta involves the following steps: First, aortic tissue is collected and fixed in 4% paraformaldehyde (PFA), followed by paraffin embedding. Tissue sections (4–6 µm) are mounted on adhesive slides. After dewaxing with xylene and rehydrating through a gradient of ethanol, antigen retrieval is performed using EDTA buffer (pH 9.0). Next, 5% goat serum is used for blocking, and the primary antibody (Protein-tech, 11600-1-AP, Wuhan, China) is applied in a diluted form, followed by incubation at 4 °C overnight. After washing, a secondary antibody labeled with HRP is incubated at room temperature for 1 h. The staining is visualized using DAB, and nuclear counterstaining with hematoxylin can be performed. Finally, tissue sections are dehydrated and mounted, and the HCK-positive cells are observed under a microscope (Olympus, Tokyo, Japan), evaluating the staining intensity and distribution.

### 2.15. Quantitative Real-Time Polymerase Chain Reaction (q-RTPCR) Experiment

After collecting anticoagulated peripheral blood from the mice, erythrocytes were lysed to isolate white blood cells. The aortas were then harvested and homogenized, and total RNA was extracted using an RNA extraction kit (Vazyme, R401, Wuhan, China). The RNA concentration and purity were assessed using a spectrophotometer (ThermoFisher Scientific, MA, USA). Subsequently, the RNA was reverse-transcribed into complementary DNA (cDNA) using a reverse transcription reagent. A quantitative PCR (qPCR) was performed to amplify the target genes, with GAPDH serving as an internal control for normalization. The cycling conditions for the qPCR were as follows: (1) initial denaturation at 95 °C for 2 min to activate the DNA polymerase, (2) 40 amplification cycles, (3) denaturation at 95 °C for 15 s, (4) annealing at 65 °C for 20–30 s, and (5) extension at 72 °C for 30 s. Fluorescence signals were monitored in real time during the amplification process to determine the cycle threshold (Ct) values. The relative expression of the target genes was calculated using the ΔΔCt method or a standard curve, with expression normalized to the internal control gene, and differences in gene expression levels were compared across different samples. The primers used for qPCR amplification were *HCK*: forward 5′-TCGTTGTCTGTTCGAGACTTTG, reverse 5′-TCTTGTAGTGGAGCACGAGTT; *NCKAP1L*: forward 5′-TGTCCGAAATAGCACGCAACA, reverse 5′-ATCCCGAAATTCCATGACATCC; *WAS*: forward 5′-CCAGCCGTTCAGCAGAACAT, reverse 5′-GGTTATCCTTCACGAAGCACA; *GAPDH*: forward 5′-AGGTCGGTGTGAACGGATTTG, reverse 5′-TGTAGACCATGTAGTTGAGGTCA.

### 2.16. Statistical Analysis

Statistical analysis was conducted using GraphPad (version 9.0.3) and R software (version 4.2.3). The comparison of biomarker expression levels between stable and unstable plaques, as well as the assessment of immune cell proportions, immune scores, and consensus clustering, was performed using the Kruskal–Wallis test. The correlation analyses between immune cells, Immune Scores, and biomarkers were conducted using Spearman correlation analysis. A *p* value < 0.05 was considered statistically significant.

## 3. Results

### 3.1. Selection of Gene Modules Associated with Periodontitis

**Figure 2:**
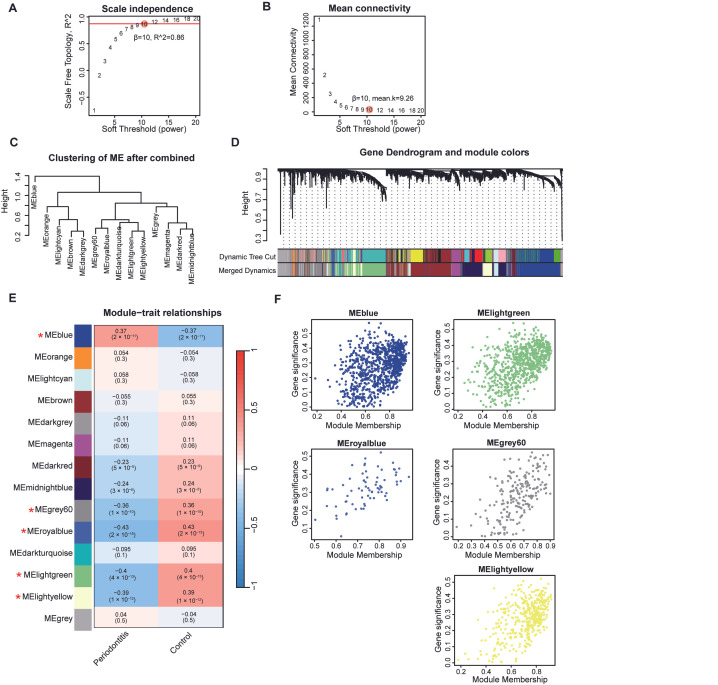
Gene modules associated with periodontitis. The optimal soft-thresholding power (10) was determined based on the scale-free topology (**A**) and average connectivity (**B**). (**C**) Modules with a similarity > 0.75 were merged and hierarchy clustering performed. (**D**) The dynamic tree illustrates each module after merging. (**E**) A heatmap illustrates the correlation between gene modules and clinical features, along with the corresponding *p*-values. Modules with a correlation coefficient > 0.3 and a *p*-value < 0.05 are identified as periodontitis-related gene modules and marked with asterisks (*). (**F**) Scatter plots display the correlation between module membership and the gene significance of periodontitis-related gene modules.

Firstly, we applied the WGCNA to identify gene expression modules related to periodontitis. Based on the scale-free topology and mean connectivity, we selected a robust soft threshold as 10 (Figure 2A,B). After merging modules with a similarity > 0.75, we performed hierarchical clustering on the resulting modules (Figure 2C). The dynamic tree cut illustrates each module and its corresponding color after merging (Figure 2D). A heatmap depicts the relationship between each gene expression module and periodontitis (Figure 2E, Supplementary Table S1). We defined modules with a correlation coefficient > 0.3 and a *p* value < 0.05 as periodontitis-related gene expression modules, visualizing the gene weights and inter-module relationships using scatter plots (Figure 2F).

### 3.2. Different Gene Expression Patterns in Stable and Unstable Atherosclerotic Plaques

**Figure 3:**
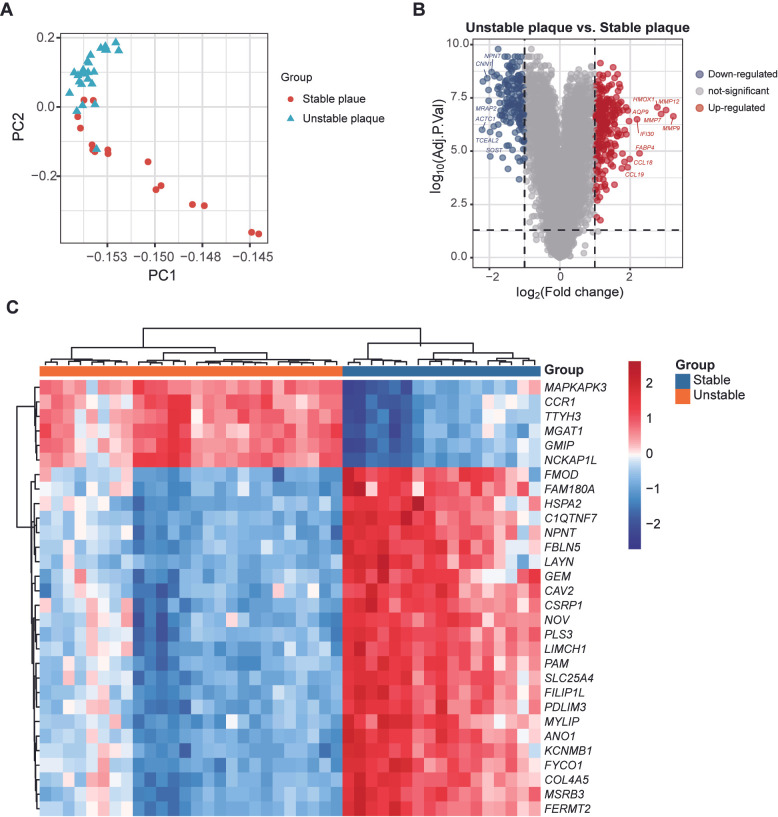
Differential gene expression between stable and unstable plaques. (**A**) The PCA plot illustrates the overall differences in gene expression after dimensionality reduction between stable and unstable plaques. (**B**) The volcano plot displays the differentially expressed genes between stable and unstable plaques. (**C**) The heatmap indicates the expression levels of the top 30 statistically significant genes in both stable and unstable plaques.

Next, we assessed the differential gene expression between stable and unstable plaques in the discovery dataset GSE163154. The PCA plot revealed distinct differences in the overall gene expression patterns between the two groups (Figure 3A). We set thresholds of |Log2FC| > 1 and an adjusted *p* value < 0.05. Compared to stable plaques, 249 genes were found to be upregulated and 187 genes downregulated in unstable plaques (Figure 3B). The full result of DEGs between stable and unstable atherosclerotic plaque is demonstrated in Supplementary Table S2. A heatmap illustrates the expression levels of the top 30 statistically significant genes in both sample groups (Figure 3C).

### 3.3. Functional Enrichment and PPI Network of Periodontitis-Related DEGs

**Figure 4:**
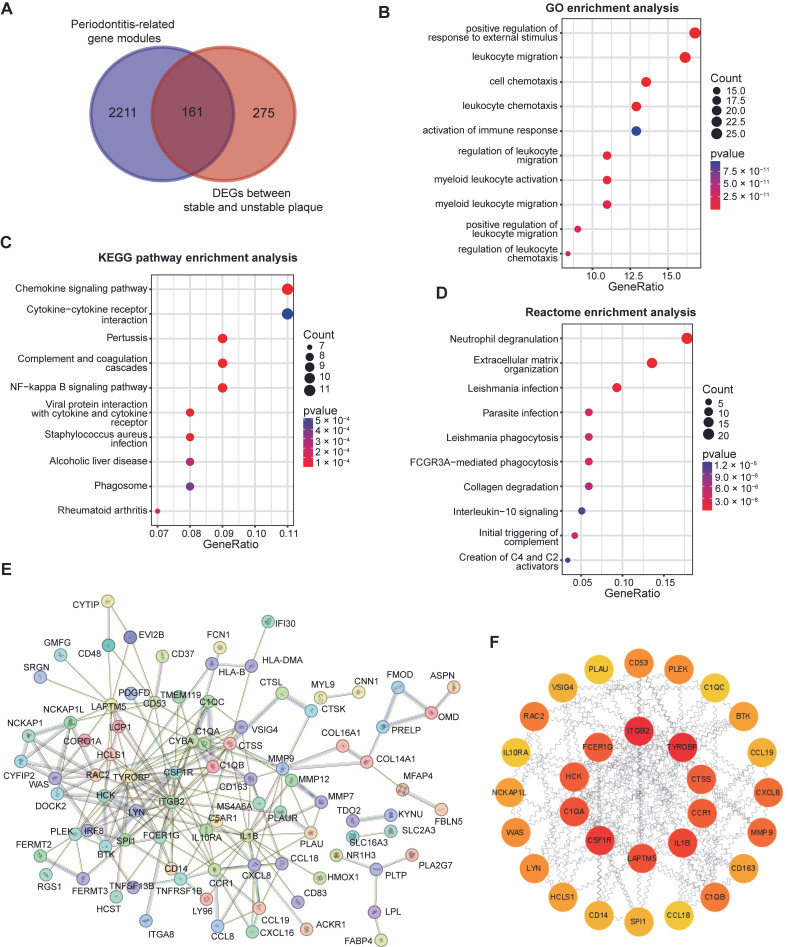
Functional enrichment analysis and PPI network of periodontitis-related DEGs. (**A**) The Venn diagram reveals a total of 161 genes identified as periodontitis-related DEGs. Functional enrichment analysis of periodontitis-related DEGs was conducted using GO (**B**), KEGG (**C**), and Reactome databases (**D**), highlighting the top ten statistically significant pathways. (**E**) The protein–protein interaction network for the 161 differential genes associated with periodontitis. (**F**) The top 30 node genes ranked with the Degree algorithm of CytoHubba plug.

We identified 161 intersecting genes by taking the intersection of genes from periodontitis-related modules and DEGs between stable and unstable plaque (Figure 4A, Supplementary Table S3). These genes were defined as periodontitis-related DEGs. To elucidate the specific roles of periodontitis-related DEGs in the destabilization of atherosclerotic plaques, we conducted functional enrichment analyses using the GO, KEGG, and Reactome databases. The top ten most significantly enriched pathways were selected for presentation (Supplementary Tables S4–S6). GO enrichment analysis revealed the enhanced reactivity of immune cells, with leukocyte migration and chemotaxis likely mediating the impact of periodontitis on atherosclerotic stability (Figure 4B). Similarly, KEGG enrichment analysis indicated the involvement of chemokines and cytokines (Figure 4C), whereas Reactome enrichment analysis highlighted not only neutrophil degranulation and the generation of complement and chemokines but also suggested periodontitis-related DEGs involved in the degradation of fibrin and extracellular matrix, which may play a crucial role in the instability of atherosclerotic plaques (Figure 4D).

Subsequently, we utilized the STRING database to illustrate the interaction network among these 161 proteins, resulting in a network consisting of 88 nodes and 209 edges (Figure 4E). To identify key nodes for further analysis, we employed the CytoHubba plugin in Cytoscape, utilizing the Degree algorithm to select the top 30 core genes and their potential connection (Figure 4F).

### 3.4. Identification of Biomarkers for Unstable Plaque Associated with Periodontitis

**Figure 5:**
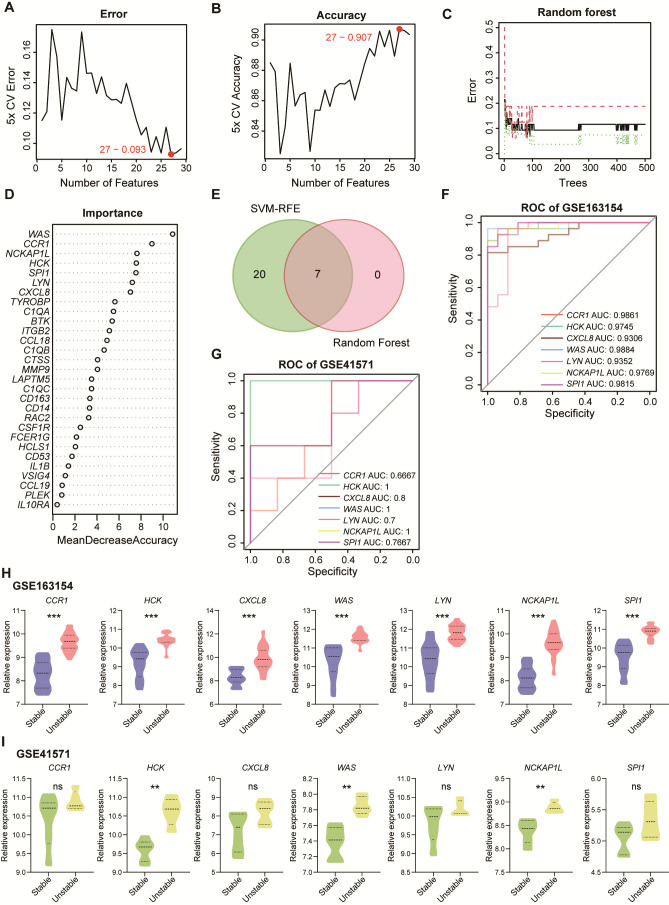
Selection of biomarkers for unstable plaques associated with periodontitis. SVM-REF method-demonstrated DEGs with the lowest error (**A**) and highest accuracy (**B**) after 100 folds were considered the most suitable candidates. (**C**) Visualization of diagnostic errors related to random forests for stable plaques, unstable plaques, and all groups. The red line represents the upper bound of the random forest model, which is the maximum value of the error; the black line represents the average value of the model error; and the green line indicates the lower bound of the model error, which is the minimum value of the error. (**D**) The importance scores calculated from the random forest analysis were used to rank 30 node genes based on importance. (**E**) Venn diagram illustrates the identification of a total of 7 candidate biomarkers. The ROC curves display the AUC for the 7 candidate biomarkers in the discover dataset GSE163154 (**F**) and the validation dataset GSE41571 (**G**). Expression of the 7 biomarkers in discover dataset GSE163154 (**H**) and the validation dataset GSE41571 (**I**), comparing the expression of candidate biomarkers in stable and unstable plaques. Kruskal–Wallis test in (**H**,**I**). *p* < 0.05; **, *p* < 0.01; ***, *p* < 0.001; ns, not significant.

To select key biomarkers from the 30 core genes, we employed a series of machine learning algorithms. Initially, the SVM-RFE algorithm indicated that 27 genes yielded the lowest error rate and highest accuracy for diagnosing plaque progression (Figure 5A,B). Subsequently, we applied the random forest algorithm and identified the top seven important genes based on accuracy (Figure 5C,D). By taking the intersection of the two algorithms, we identified seven candidate biomarkers: *CCR1*, *HCK*, *CXCL8*, *WAS*, *LYN*, *NCKAP1L*, and *SPI1* (Figure 5E, Supplementary Table S7). Then we constructed ROC curves for these candidates in discovery and validation datasets to calculate the AUC (Figure 5F,G). Furthermore, the expression levels of these seven candidate biomarkers across the two datasets were compared (Figure 5H,I). Following the criteria of an AUC > 0.7 and significant expression differences in datasets GSE613451 and GSE41571, we ultimately identified *HCK*, *NCKAP1L*, and *WAS* as biomarkers for unstable atherosclerotic plaques associated with periodontitis.

### 3.5. Nomogram for Diagnosing Unstable Plaques and Single-Gene GSEA of Biomarkers

**Figure 6:**
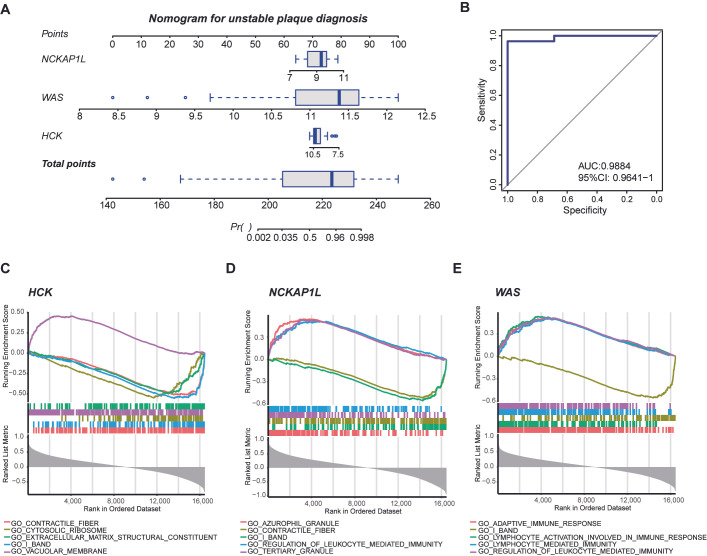
Diagnostic nomogram and single-gene GSEA for biomarkers of unstable plaques associated with periodontitis. (**A**) The nomogram for diagnosing unstable plaques was constructed based on the expression levels of *HCK, NCKAP1L*, and *WAS*. (**B**) The ROC curve illustrates the effectiveness of the nomogram in predicting unstable plaques. Single-gene GSEA analysis of the diagnostic markers *HCK* (**C**), *NCKAP1L* (**D**), and *WAS* (**E**) reveals their potential functions in unstable plaques.

In clinical practice, identifying high-risk unstable atherosclerotic plaques is crucial for preventing acute cardiovascular and cerebrovascular events. To further evaluate the diagnostic efficacy of periodontitis-related biomarkers in unstable plaques, we constructed a nomogram (Figure 6A). The associated ROC curve demonstrates its high diagnostic efficacy (AUC: 0.9884) (Figure 6B). The single-gene GSEA provided insights into the potential roles of each biomarker within unstable plaques (Supplementary Tables S8–S10). Notably, *HCK* appears to be associated with fibrotic activity, ribosome biogenesis, and extracellular matrix formation (Figure 6C). In contrast, *NCKAP1L* and *WAS* play significant roles in regulating immune system activities (Figure 6D,E).

### 3.6. Immune Infiltration Landscape of Atherosclerotic Plaques

**Figure 7:**
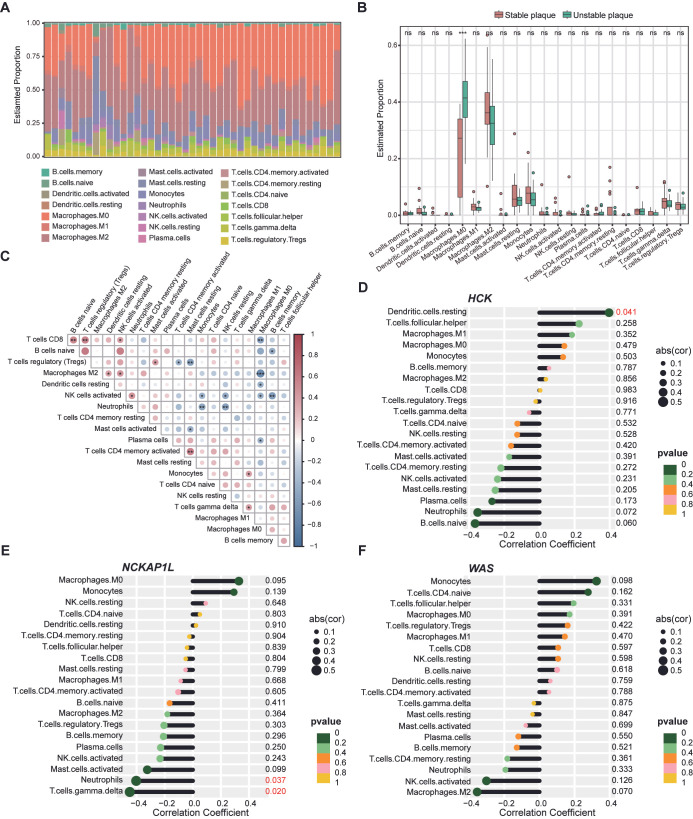
Immune infiltration analysis of stable and unstable plaques. (**A**) The bar chart displays the proportion of immune cell composition for each sample in both the stable and unstable plaque groups. (**B**) The box plot compares the differences in immune cell proportions between the stable and unstable plaque groups. (**C**) The heatmap illustrates the correlations between the proportions of immune cells within the unstable plaque group. The size of the circles represents the magnitude of the absolute value of the correlation coefficient. The correlation between relative expression of *HCK* (**D**), *NCKAP1L* (**E**), and *WAS* (**F**) and the composition of immune cells in unstable plaques is shown. Cell types with a *p* value < 0.05 are marked in red. Kruskal–Wallis test in (**B**) and Spearman correlation test in (**C**–**F**). *, *p* < 0.05; **, *p* < 0.01; ***, *p* < 0.001, ns means not significant.

The gene enrichment analyses indicate that immune inflammation mediates the impact of periodontitis on atherosclerotic plaques, prompting us to examine the immune cell composition in different states of atherosclerotic plaques. Using the CIBERSORT R package, we calculated the proportion of different immune cells in plaque samples (Supplementary Table S11). A bar plot reveals the relative proportions of immune cell types in each plaque sample within the discovery dataset (Figure 7A). Notably, a comparison of immune cell abundance between stable and unstable plaques shows a significant increase in M0 macrophages in unstable plaques, suggesting their potential role in promoting plaque instability (Figure 7B). A heatmap illustrates the correlations among various immune cells within unstable plaques, indicating possible intercellular regulatory interactions (Figure 7C). Further analysis correlating the expression of periodontitis-related biomarkers with immune cell composition reveals a significant relationship between *HCK* and dendritic cells (DCs), while *NCKAP1L* is significantly correlated with gamma delta T cells and neutrophils (Figure 7D–F).

**Figure 8:**
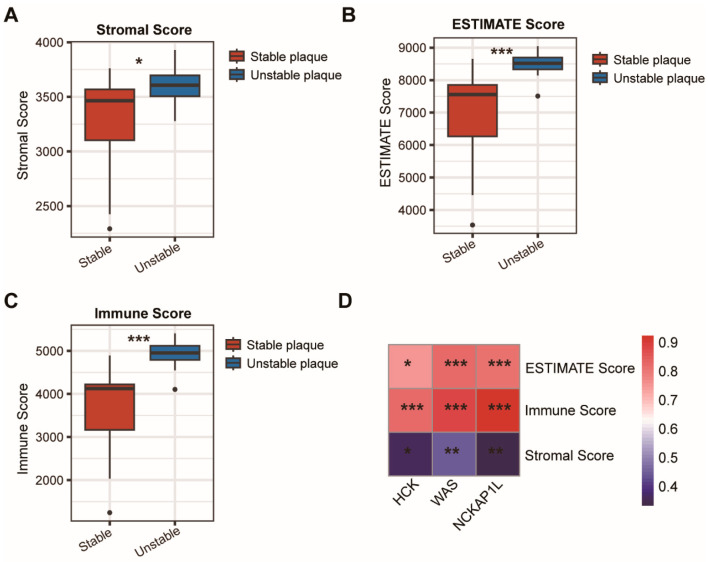
Comparison of immune microenvironment in stable and unstable plaques. Different Stromal Score (**A**), ESTIMATE Score (**B**), and Immune Score (**C**) between the stable plaque and the unstable plaque. (**D**) Heatmap demonstrates the correlation between the expression of *HCK*, *WAS*, and *NCKAP1L* with Stromal Score, ESTIMATE Score, and Immune Score. Kruskal–Wallis test in (**A**–**C**). Spearman correlation test in (**D**). *, *p* < 0.05; **, *p* < 0.01; ***, *p* < 0.001.

Additionally, we employed the ESTIMATE algorithm to assess the Stromal Score, ESTIMATE Score, and Immune Score in stable and unstable plaques. The results show a significant elevation of the three scores in unstable plaques, highlighting more pronounced changes in immune inflammation and stromal components (Figure 8A–C). The heatmap demonstrates the relationships between the expression of periodontitis-related biomarkers and these three immune scores, revealing a significant negative correlation between Stromal Scores and biomarker expression (Figure 8D).

### 3.7. Characterization of Biomarkers’ Expression in Single-Cell mRNA Data of Unstable Plaques

**Figure 9:**
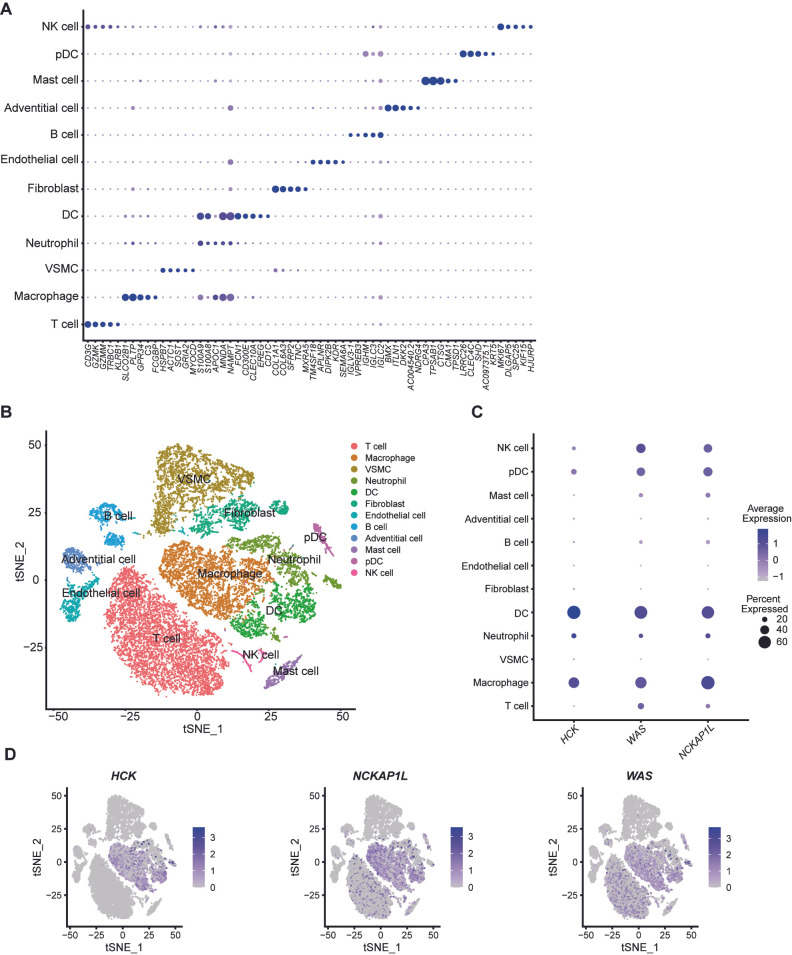
Single-cell mRNA characterization of unstable plaques. (**A**) Dot plot illustrates the differentiation of cells within unstable plaques into 12 subpopulations based on the expression of characteristic genes, with annotations provided for each subpopulation. The size of the dots represents the relative expression level of the gene across different cell types. (**B**) t-SNE dimensionality reduction reveals the expression profiles of 20,687 cells obtained from 6 unstable plaques, categorized into 12 distinct cell subpopulations. Dot plot (**C**) and feather plot (**D**) depict the expression levels of biomarkers *HCK*, *NCKAP1L*, and *WAS* across different cell types within the unstable plaques.

To further investigate the expression of periodontitis-related biomarkers in various cell types within unstable plaques, we analyzed single-cell mRNA data from six unstable plaque specimens. Based on the expression of characteristic genes in each cell cluster, we identified and annotated a total of 12 distinct cell populations (Figure 9A, Supplementary Table S12). The analysis yielded expression data for 20,687 cells, which we visualized using t-SNE dimensionality reduction (Figure 9B). The dot plot and feather plot clearly illustrate the expression of *HCK*, *NCKAP1L*, and *WAS* within the plaques (Figure 9C,D). Notably, *HCK* is predominantly expressed in macrophages and DCs, with a smaller proportion found in neutrophils. In contrast, *NCKAP1L* and *WAS* exhibit similar expression patterns, primarily localized to macrophages, DCs, NK cells, and T cells. These expression patterns align with the findings from the single-gene GSEA analysis, suggesting their potential roles in immune cell function.

### 3.8. Potential Subtypes in Unstable Plaques

**Figure 10:**
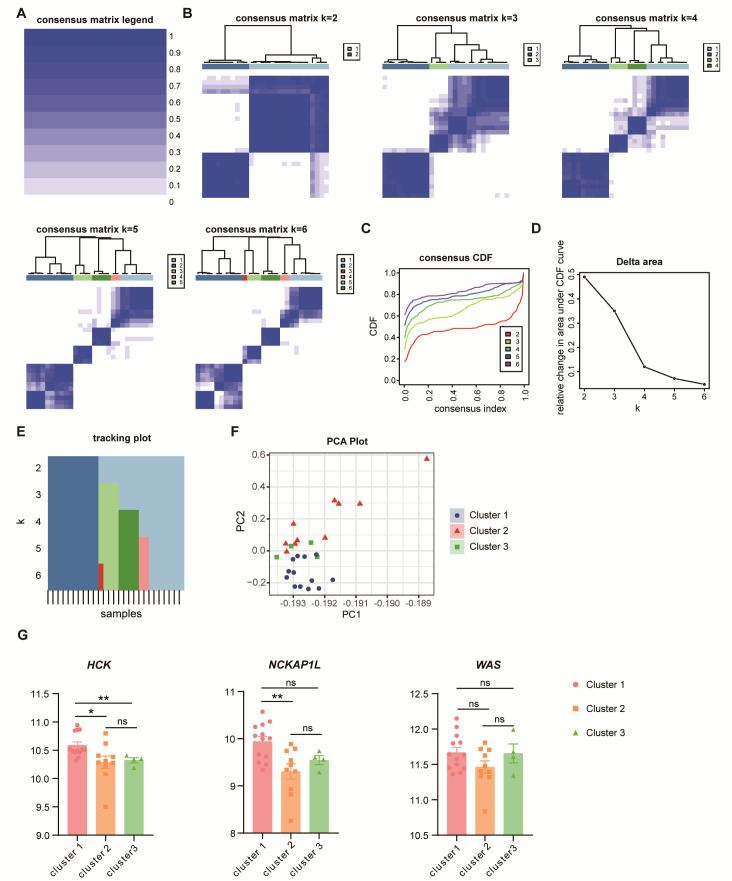
Consensus clustering of unstable plaques. (**A**) Heatmap illustrates the correlation matrix obtained from the consensus clustering analysis. (**B**) The consensus cluster heatmap for K-values ranging from 2 to 6 are displayed. CDF curves (**C**) and the corresponding AUC (**D**) are presented for K-values of 2 to 6. (**E**) A tracking plot shows the sample assignments at K-values of 2 to 6. The color of the modules represents the grouping of samples under different values of k. (**F**) PCA depicts the distribution of samples from 3 clusters. (**G**) Different expression of *HCK*, *NCKAP1L*, and *WAS* across the three identified subgroups is shown. CDF, Cumulative Distribution Function. Kruskal–Wallis test in (**G**) *, *p* < 0.05; **, *p* < 0.01; ns, not significant.

Unstable atherosclerotic plaques exhibit various pathological features, including ruptured plaques, erosive plaques, and partially calcified nodular lesions [8]. To investigate the expression patterns of potential subtypes within unstable plaques, we employed consensus clustering methods. The clustering heatmap illustrates the results of K-means clustering for values of K ranging from 2 to 6 (Figure 10A,B). After a careful evaluation of the Cumulative Distribution Function (CDF) and the corresponding area under the CDF, we determined K = 3 as the optimal clustering criterion (Figure 10C,D). The tracking plot displays the sample assignments across different K-values (Figure 10E). At K = 3, we observed a distinct clustering of the samples (Figure 10F). Notably, the expression levels of *HCK* and *NCKAP1L* varied among the different subgroups, indicating their potential roles in the various pathological types of lesions (Figure 10G).

### 3.9. Validation of Periodontitis-Associated Atherosclerotic Biomarker Expression

**Figure 11:**
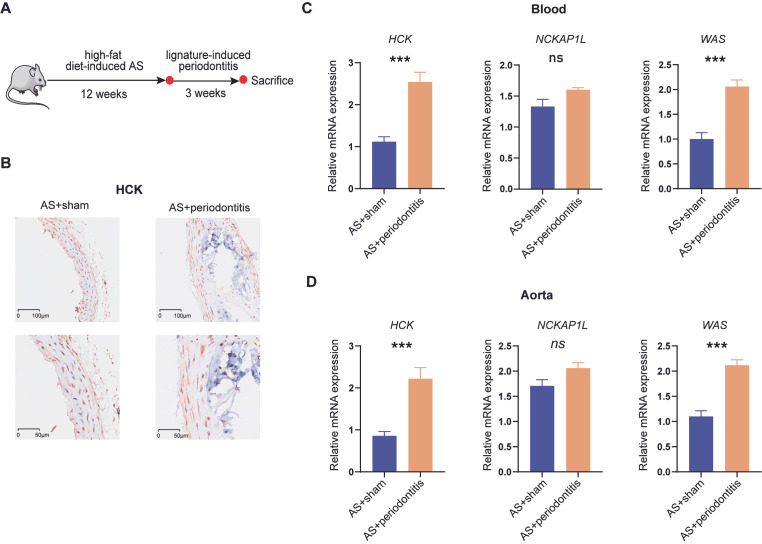
Validation of mouse comorbidity model. (**A**) Schematic representation of mouse model induction. (**B**) Immunohistochemical analysis showing differential expression of *HCK* in atherosclerotic plaques with or without chronic periodontitis in mice. Expression of periodontitis-associated biomarkers in (**C**) blood and (**D**) aorta (n = 5). Students’ t test in (**C**,**D**). AS, atherosclerosis. ***, *p* < 0.001; ns, not significant.

To validate the expression of biomarkers in the dual disease model, periodontitis was introduced into the atherosclerotic mouse model, as depicted in Figure 11A. Immunohistochemical analysis of the aorta revealed a significant increase in the expression level of *HCK* in atherosclerotic mice with periodontitis (Figure 11B). Further transcriptional-level validation experiments suggested the robustness of *HCK* and *WAS* as periodontitis-associated biomarkers for atherosclerosis, while the expression of *NCKAP1L* showed no significant differences (Figure 11C,D).

## 4. Discussion

Atherosclerosis serves as the pathophysiological basis for coronary artery disease (CAD) and ischemic stroke, presenting a notably high incidence and mortality rate globally [36]. Stable atherosclerotic plaques typically manifest clinically as chronic ischemic syndromes, while unstable plaques are associated with ischemic strokes and acute cardiovascular events, thus drawing significant attention [36,37]. Unstable plaques are often characterized by a thin fibrous cap and a necrotic core [37]. Epidemiological studies have indicated a correlation between periodontitis disease and the progression of carotid plaques, as well as an increased incidence of acute cardiovascular events [9]. Recent studies have further highlighted the genetic and inflammatory links between periodontitis and systemic conditions such as atherosclerosis, underscoring the importance of shared pathways in disease progression [38,39].

This suggests that periodontitis may contribute to the instability of atherosclerotic plaques. The mechanisms by which periodontitis exacerbates atherosclerosis may involve two primary pathways: (1) chronic inflammation—periodontitis is characterized by chronic inflammation of the gums, leading to the release of inflammatory factors into the bloodstream, which promotes the development and progression of atherosclerosis [16]; (2) bacterial dissemination—bacteria associated with periodontitis can enter the bloodstream through inflamed gum tissue, potentially contributing to plaque formation and progression [40]. However, the precise mechanisms by which periodontitis leads to the progression of atherosclerosis, along with the related diagnostic biomarkers, remain to be elucidated further. In our study, we utilize transcriptomic data from public datasets concerning localized gingival tissues from periodontitis and unstable atherosclerotic plaques. We identify the potential mechanisms and biomarkers linking periodontitis to atherosclerotic plaque vulnerability, construct a diagnostic model, and clarify the roles of these biomarkers within the immune cells of unstable plaques, which provides novel theoretical support for the diagnosis and treatment of unstable atherosclerotic plaques.

Previously published study investigated the role of periodontitis in the pathogenesis of atherosclerosis by comparing gene expression patterns between chronic periodontitis and healthy gingival tissues, as well as between atherosclerotic plaque and normal arterial tissues [41]. Numerous clinical studies have confirmed that unstable atherosclerotic plaques are critical factors in acute cardiovascular and cerebrovascular events [7]. Additionally, periodontitis has been positively correlated with carotid intima–media thickness, indicating a higher risk of acute cardiovascular events [12,13]. These findings suggest that chronic periodontitis plays a significant role in the progression of atherosclerotic plaques. However, its underlying mechanisms and therapeutic targets remain unclear. Our study is the first to investigate the immuno-inflammatory mechanisms and key therapeutic targets involved in chronic periodontitis-induced atherosclerotic plaque instability, which may provide crucial theoretical support for clinical diagnosis and intervention regarding high-risk unstable atherosclerotic plaques.

By intersecting the genes associated with periodontitis and DEGs in unstable plaques, we identified 161 periodontitis-related DEGs. Enrichment analysis revealed that these genes are primarily involved in the activation of immune inflammatory cells, the secretion of chemokines and cytokines, and the degradation of the collagen matrix. Previous studies have highlighted the presence of a complex microenvironment within atherosclerotic plaques, encompassing processes such as foam cell formation, the migration of VSMCs and their transition from a contractile to a synthetic phenotype, extracellular matrix remodeling, plaque growth, and fibrous cap formation [2,8,37]. In these processes, inflammation plays a pivotal role. Elevated inflammation levels are closely associated with the increased activation of immune cells, which, through the release of inflammatory factors, contribute to the expansion of the necrotic core within the plaque, thereby reducing its stability [42,43]. Research indicates that inflammatory cells in the periodontitis lesions can enter the bloodstream via lymphatic vessels, subsequently infiltrating the plaque and causing a disruption in the homeostasis of atherosclerosis, which induces instability in atherosclerotic plaques [16].

By screening the node genes of the PPI network and applying various machine learning methods, we identified *HCK*, *NCKAP1L*, and *WAS* as biomarkers of periodontitis-related unstable plaques. The gene *HCK* encodes hematopoietic cell kinase, a member of the non-receptor protein tyrosine kinase family, expressed in cells of the bone marrow and B lymphocyte lineage, which regulates numerous cell signaling pathways and biological effects [44]. Previous studies have indicated that the hyperactivation of HCK is linked to various immune inflammatory and tumor-related diseases [44]. Its activation not only enhances the secretion of growth factors and pro-inflammatory cytokines in bone marrow cells but also promotes the inflammatory polarization of macrophages, their migration, and the degranulation and cytotoxic activity of neutrophils [45]. In human macrophages, increased *HCK* expression augments the transcription of TNFα and IL-6 induced by TLR4 through the AP-1 transcription factor complex, significantly enhancing macrophage responses to LPS [44,46]. Notably, *HCK* has been recognized as a hub gene in advanced atherosclerotic plaques, where its knockout can enhance plaque stability by reducing monocyte recruitment and activity within the plaque [47]. Our research suggests that *HCK* is a critical molecule in the instability of atherosclerotic plaques induced by periodontitis, with an elevated expression in unstable plaques mediating the degradation of the fibrous matrix, which, combined with previous studies, underscores the important role of *HCK* in regulating plaque stability.

*NCKAP1L* encodes the NCK-associated protein 1-like gene, also known as hematopoietic protein 1, which serves as a lineage-specific regulator of the actin cytoskeleton [48]. NCKAP1L plays a vital role in the activation, migration, and cell–cell interactions of lymphocytes and bone marrow cells [49]; its overexpression is often associated with sustained immune activation and the development of autoimmune inflammatory diseases [48]. Previous studies have demonstrated that mutations in *NCKAP1L* can impair actin polymerization, synapse formation, and immune cell migration [50,51]. The knockout of *NCKAP1L* has been shown to block T cell proliferation and effector function [52]. Our findings indicate that *NCKAP1L* expression is elevated in unstable plaques, and the single-gene GSEA reveals its involvement in the immune regulation of leukocytes, suggesting a link to increased inflammation levels in vulnerable plaques.

*WAS* encodes the Wiskott–Aldrich Syndrome Protein (WASP), a member of the actin nucleation-promoting factor family, which is widely expressed in non-erythroid hematopoietic cells [53]. WASP has been identified as a crucial regulator of the actin cytoskeleton in hematopoietic cells, playing significant roles in lymphocyte and myeloid cell migration, receptor signaling, cytotoxicity, and phagocytosis [53,54,55,56]. Studies indicate that WASP is involved in T cell development and intrinsic functions through both actin-dependent and -independent mechanisms, impacting processes such as cell proliferation, differentiation, and survival [57]. Deficiencies in WASP lead to various effects in the adaptive immune system, including the overexpression of TH2 cells, the hyperactivation of CD8^+^ T cells, the reduction of T regulatory cells, and increased TH17 cells, along with elevated pro-inflammatory macrophages and cytokines [58]. Recent findings have also demonstrated that WASP plays a role in the transcriptional and epigenetic regulation of myeloid cells [59]. These insights align with our research, highlighting the critical involvement of WASP in modulating immune responses and potentially contributing to the instability of atherosclerotic plaques.

Functional enrichment analysis revealed the critical role of immune inflammation in the destabilization of atherosclerotic plaques. Consequently, we conducted a comprehensive evaluation of immune cell infiltration in unstable atherosclerotic plaques. Our findings demonstrated a significant increase in the relative proportion of M0 macrophages within unstable plaques, underscoring the pivotal role of macrophages in plaque progression. Previous studies have indicated that macrophages are the predominant immune cells within plaques, capable of inducing dynamic changes in atherosclerotic lesions [37]. In the complex microenvironment of plaques, macrophages can polarize into pro-inflammatory M1 or anti-inflammatory M2 phenotypes [60]. In unstable plaques, macrophages directly contribute to the expansion of the necrotic core and thinning of the fibrous cap, thereby increasing the risk of rupture [61]. In a clinical study of symptomatic carotid plaques (n = 526), the abundance of macrophage infiltration was closely associated with plaque instability, indicating that macrophages play a crucial role in plaque pathology [62]. With the rapid advancement of single-cell technologies, identifying the macrophage subpopulations associated with plaque rupture and their underlying mechanisms has become essential. In this study, we employed single-cell mRNA analysis to visually elucidate the expression patterns of *HCK, NCKAP1L,* and *WAS* in various cell types within unstable plaques. This finding aligns with our previous discussions and provides a theoretical basis for targeting these genes in diagnostic and therapeutic interventions.

Our study is the first to investigate the potential mechanisms by which periodontitis contributes to the instability of atherosclerotic plaques. By utilizing public datasets, we explored not only the pathways leading to plaque destabilization due to periodontitis but also identified specific biomarkers and associated cell subpopulations, which provides evidence for the progression of atherosclerotic plaques induced by periodontitis and offers targeted genes and cells for diagnosis and intervention. However, several limitations should be acknowledged. First, the issue of sample size arises. Although GSE163451 includes samples from 27 unstable and 16 stable carotid plaques, a larger sample size would enhance the robustness of our conclusions. Second, our findings are based on bioinformatics analyses and require validation through clinical studies or animal models. Due to ethical constraints, obtaining specimens of atherosclerotic plaques is challenging, limiting our ability to conduct further validation. Additionally, in animal models, plaques formed in *APOE* or *LDLR* knockout mice fed a high-fat diet tend to be stable plaque, but the methods for establishing models of unstable atherosclerotic plaques remain underdeveloped [63]. Therefore, more foundational research is needed to verify our conclusions.

## 5. Conclusions

This study leveraged transcriptomic data to investigate how periodontitis promotes the transition of stable atherosclerotic plaques toward instability through enhanced immune inflammation and the degradation of fibrous matrices. We identified *HCK*, *NCKAP1L*, and *WAS* as key diagnostic biomarkers for periodontitis-related unstable plaques and developed a diagnostic nomogram to facilitate clinical risk assessment. Immune infiltration analysis revealed the central role of M0 macrophages in plaque destabilization and characterized the expression patterns of biomarkers across various immune cell types. Furthermore, we constructed regulatory networks involving transcription factors, miRNAs, and drug interactions, uncovering potential therapeutic targets for intervention. These findings advance our understanding of the molecular mechanisms linking periodontitis to cardiovascular pathology, highlighting the interplay between immune inflammation and extracellular matrix remodeling in plaque instability. By integrating multi-omics data and computational analyses, this study provides a framework for identifying diagnostic biomarkers and therapeutic strategies, offering new avenues for managing the systemic consequences of periodontal disease.

## Figures and Tables

**Table 1:** Transcriptomic datasets used in this study.

GSE Number	Platform	PMID	Samples	Source Types	Note
GSE16134	GPL570	24646639	240 Periodontitis and 70 Control	Gingival tissue	Test dataset
GSE163154	GPL6104	37920458	16 Stable plaque and 27 Unstable plaque	Carotid plaque	Test dataset
GSE41571	GPL570	23122912	6 Stable plaque and 5 Unstable plaque	Carotid plaque	Validation dataset
GSE253904	GPL24676	38385291	6 Unstable plaque	Carotid plaque	Validation dataset

## Data Availability

The datasets utilized in this study are openly accessible and were obtained from the GEO (https://www.ncbi.nlm.nih.gov/geo/) (accessed on 11 March 2024). Four datasets were used, including GSE16134 (https://www.ncbi.nlm.nih.gov/geo/query/acc.cgi?acc=GSE16134) (accessed on 11 March 2024), GSE163154 (https://www.ncbi.nlm.nih.gov/geo/query/acc.cgi?acc=GSE16154) (accessed on 11 March 2024), GSE41571 (https://www.ncbi.nlm.nih.gov/geo/query/acc.cgi?acc=GSE41571) (accessed on 11 March 2024), GSE253904 (https://www.ncbi.nlm.nih.gov/geo/query/acc.cgi?acc=GSE253904) (accessed on 11 March 2024).

## Supplementary Materials

The following supporting information can be downloaded at: https://www.mdpi.com/article/10.3390/cimb47030197/s1.

## Abbreviations

AUC	area under the curve
CAD	coronary artery disease
CI	confidence interval
CDF	Cumulative Distribution Function
DEG	differentially expressed genes
DCs	dendritic cells
FC	fold change
GEO	Gene Expression Omnibus
GO	Gene Ontology
GSEA	gene set enrichment analysis
KEGG	Kyoto Encyclopedia of Genes and Genomes
miRNA	micro RNA
MMPs	matrix metalloproteinases
PPI	protein–protein interaction
ROC	receiver operating characteristics
SVM-RFE	support vector machine recursive feature elimination
TCR	T cell receptor
TLR	toll-like receptor
TOM	Topological Overlap Matrix
WGCNA	weighted genes co-expression network analysis
VSMCs	vascular smooth muscle cells
